# Expression of Annexin-A1 and Galectin-1 Anti-Inflammatory Proteins and mRNA in Chronic Gastritis and Gastric Cancer

**DOI:** 10.1155/2013/152860

**Published:** 2013-01-31

**Authors:** Yvana Cristina Jorge, Mayra Mioto Mataruco, Leandro Pires Araújo, Ana Flávia Teixeira Rossi, Juliana Garcia de Oliveira, Marina Curado Valsechi, Alaor Caetano, Kenji Miyazaki, Célia Sebastiana de Jesus Fazzio, Jorge Alberto Thomé, Paula Rahal, Sonia Maria Oliani, Ana Elizabete Silva

**Affiliations:** ^1^Department of Biology, São Paulo State University (UNESP), Campus de São José do Rio Preto, Rua Cristóvão Colombo 2265, 15054-000 São José do Rio Preto, SP, Brazil; ^2^Rio Preto Endoscopy Center, São José do Rio Preto, SP, Brazil; ^3^Endoscopy Service, Hospital de Base, São José do Rio Preto, SP, Brazil; ^4^Legal Medicine Department and Pathology Service, Hospital de Base, São José do Rio Preto, SP, Brazil; ^5^Institute of Anatomical Pathology and Cytopathology, São José do Rio Preto, SP, Brazil

## Abstract

*Objective*. The anti-inflammatory proteins annexin-A1 and galectin-1 have been associated with tumor progression. This scenario prompted us to investigate the relationship between the gene and protein expression of annexin-A1 (*ANXA1*/AnxA1) and galectin-1 (*LGALS1*/Gal-1) in an inflammatory gastric lesion as chronic gastritis (CG) and gastric adenocarcinoma (GA) and its association with *H. pylori* infection. *Methods*. We analyzed 40 samples of CG, 20 of GA, and 10 of normal mucosa (C) by the quantitative real-time PCR (qPCR) technique and the immunohistochemistry assay. *Results*. High *ANXA1* mRNA expression levels were observed in 90% (36/40) of CG cases (mean relative quantification RQ = 4.26  ±  2.03) and in 80% (16/20) of GA cases (mean RQ = 4.38  ±  4.77). However, *LGALS1* mRNA levels were high (mean RQ = 2.44  ±  3.26) in 60% (12/20) of the GA cases, while low expression was found in CG (mean RQ = 0.43 ± 3.13; *P* < 0.01). Normal mucosa showed modest immunoreactivity in stroma but not in epithelium, while stroma and epithelium displayed an intense immunostaining in CG and GA for both proteins. *Conclusion*. These results have provided evidence that galectin-1 and mainly annexin-A1 are overexpressed in both gastritis and gastric cancer, suggesting a strong association of these proteins with chronic gastric inflammation and carcinogenesis.

## 1. Introduction

Chronic inflammation has been recognized as a process that can trigger cancer, due to the host immune response with local expression of cytokines, chemokines, adhesion molecules, and pro- and anti-inflammatory proteins that stimulate processes such as proliferation, survival, cell migration, and neovascularization. The strongest association between chronic inflammation and malignancy is observed in gastric cancer induced by *Helicobacter pylori* infection [1].

The inflammatory process resulting from *H. pylori* infection triggers a cascade of events initialized by chronic gastritis that evolves to gastric atrophy, intestinal metaplasia, dysplasia, and finally carcinoma [2]. This bacterium is present in 90% of all chronic gastritis patients [3] and in 77% of noncardia gastric cancers [4].

Both the intrinsic factors of the host and the bacterial virulence are associated with the development of gastric cancer induced by *H. pylori*. Among the bacterial genes, there is *cagA*, which is detected in about 63% of patients with gastric cancer [5]. The CagA protein is internalized by the host epithelial cells, disrupting the cell cycle and inducing cell invasion through the activation of matrix metalloproteases [6].

Bacterial lipopolysaccharides activate several cell processes, including expression of annexin-A1 (AnxA1) [7] and galectin-1 (Gal-1) [8], which are both anti-inflammatory proteins. During the inflammatory response, AnxA1 is translocated from the cell cytoplasm to the membrane, resulting in a decrease in the transmigration of inflammatory cells to the site of injury [9]. Furthermore, AnxA1 plays its anti-inflammatory role by inhibiting the activities of phospholipase A2 and inducible nitric oxide synthase [10]. It is also associated with modifications of the cytoskeleton, transport of molecules, ion flux, differentiation and migration, cell growth, and apoptosis [11].

Galectin-1 (Gal-1), a member of a family of carbohydrate-binding proteins, may act in the same manner as AnxA1 in the transmigration of inflammatory cells, being important also in T-cell apoptosis by binding to T-cell receptors, thus triggering the Faz/caspase cascade [12]. It also contributes to different events associated with carcinogenesis, including tumor transformation, cell cycle regulation, apoptosis, cell adhesion, migration, and inflammation [13, 14].

Nevertheless, there are only a few studies in the literature that evaluated the expression pattern of those anti-inflammatory mediators in gastric mucosa, but their specific functions are unclear. While in gastric cell lines the Gal-1 protein expression increased [15, 16], in gastric adenocarcinoma was observed low expression in tumor cells [17]. In turn, AnxA1 was studied in gastric tissue with discrepant findings, such as increased expression during gastric mucosal damage healing [18], loss of expression in metastatic gastric cancers [19], higher expression in diffuse-type gastric cancer compared to the intestinal type [20], and decreased expression in gastric adenocarcinoma, but with positive staining in advanced stage and peritoneal dissemination [21].

Thus, further research in this field might improve our understanding of the possible role of those anti-inflammatory proteins in carcinogenesis, which is at present fairly limited and controversial. Moreover, such studies may lead to the possible identification of AnxA1 and Gal-1 as potential biomarkers in gastric cancer progression.

Therefore, the aim of this study was to investigate the relative expression levels of *ANXA1* and *LGALS1* mRNA by quantitative real-time PCR (qPCR) and the expression of both proteins by immunohistochemical assay in inflammatory gastric lesions such as chronic gastritis compared to gastric cancer. We also investigated a possible relationship between mRNA expression levels and *H. pylori* infection and its *cagA* virulence genotype in both lesions evaluated.

## 2. Materials and Methods

### 2.1. Subjects and Samples

Gastric biopsies were obtained from seventy (70) individuals submitted to endoscopy at the Endoscopy Service of the Hospital de Base and at Rio Preto Endoscopy Center, both in São José do Rio Preto, SP, Brazil. The histopathological data were supplied, respectively, by the Legal Medicine Department and by the Pathology Service and the Institute of Anatomical Pathology and Cytopathology (IAPC), in the same city. For each individual, three (03) biopsy samples from the antrum region were collected for molecular studies and one for immunohistochemical analysis.

The gastric adenocarcinoma (GA) group comprised 20 individuals (14 male and 6 female; mean age 63.4 ± 14 years) with a histopathologically confirmed diagnosis of gastric adenocarcinoma [22]. Among the studied samples, 12 cases were diagnosed as intestinal-type adenocarcinoma (IGC) and 8 as diffuse-type adenocarcinoma (DGC). The chronic gastritis (CG) group was composed of 40 individuals (18 male and 22 female; mean age 52.5 ± 15 years) with a histopathologically confirmed diagnosis of chronic gastritis [23].

The biopsies for the control (C) group were obtained from 10 healthy individuals (7 male and 3 female; mean age 35 ± 10.8 years) with no dyspeptic gastric complaints and diagnosed as histopathologically normal gastric mucosa. Epidemiological data on the study population were collected using a standard interviewer-administered questionnaire, with questions about current and past occupation, smoking habits, alcohol intake, and family history of cancer. None of the 70 subjects were under antibiotic or anti-inflammatory treatment neither radiotherapy nor chemotherapy. About 60% of all patients in the GA group were smokers and 40% were drinkers, while all patients in the CG and C groups were nonsmokers and nondrinkers. Smokers were defined as individuals who consumed at least 100 cigarettes during their lifetime, and alcohol consumers were those who drank more than four times a week [24].

The Research Ethics Committee of the participating institution approved this research (CEP IBILCE/UNESP number 058/09), and written informed consent was obtained from all individuals studied.

### 2.2. Isolation of Total Nucleic Acids and Reverse-Transcription PCR (RT-PCR)

Soon after collection, the biopsies were stored in RNA later solution (Applied Biosystems) at −20°C, to preserve their integrity until RNA extraction. The nucleic acid extraction was performed according to the protocol accompanying the reagent TRIzol (Invitrogen) that allows the simultaneous extraction of RNA and DNA. RNA and DNA concentrations were determined in a NanoDrop ND1000 spectrophotometer (Uniscience) by measuring absorbance at 260 and 280 nm. DNA samples were stored at −20°C and used for the molecular diagnosis of *H. pylori*.

Afterwards, reverse-transcription (RT) PCR was performed in an automated thermocycler, using 2.5 *μ*g of total RNA in the presence of 1.25 *μ*L of oligo-d(T)16 (0.5 *μ*g/*μ*L), 2.0 *μ*L of RNAse Inhibitor (80 U/*μ*L), and a High Capacity cDNA Archive Kit (Applied Biosystems), in a total volume of 50 *μ*L, according to the manufacturer's instructions. The reactions were carried out for 10 minutes at 25°C, followed by 120 minutes at 37°C. The integrity of all cDNA preparations was tested by a PCR assay of a 613 bp *ACTB *(**β*-actin*) gene fragment, used as control for abundant transcripts, whose primer sequences were F: 5′-GGCATCGTGATGGACTCC-3′ and R: 3′-GCTGGAAGGTGGACAGCG-5′.

### 2.3. Molecular Diagnoses for *H. pylori*-cagA

To determine the presence of *H. pylori* infection, DNA samples were subjected to a multiplex PCR reaction containing primers for the bacterial gene *HpX *[25] and for *CYP1A1* (human housekeeping gene, which attests the integrity of the DNA). 

In summary, we used 5.0 *μ*L of 10X buffer, 5.0 *μ*L of dNTPs (1.23 mmol/L, Invitrogen), 2.0 *μ*L MgCl_2_ (25 mmol/L), 2.0 *μ*L of primers (10 nmol/*μ*L, Invitrogen), 24.5 *μ*L of dH_2_O, 5.0 *μ*L of genomic DNA, and 0.5 *μ*L of *Taq* DNA Polymerase (5 U/*μ*L, Invitrogen). The material was processed in an automated thermocycler and was initially subjected to a temperature of 94°C for 5 minutes for denaturation. Subsequently, it was subjected to 40 amplification cycles at 94°C for 45 seconds, at 60°C for 30 seconds, and at 72°C for 90 seconds, followed by a final extension cycle of 7 minutes at 72°C. The amplification products were visualized on 2.0% agarose gel stained with ethidium bromide. Fragments of 150 bp and 226 bp corresponding to genes *CYP1A1* and *HpX*, respectively, were observed.

The *H. pylori*-positive samples were then subjected to a second PCR run, to investigate the virulence genotype *cagA* of the bacterium [26]. The parameters used were the same as for the previous reaction, except the annealing temperature, which in this case was 52°C. The product was visualized on 1.0% agarose gel stained with ethidium bromide, and a 232 bp fragment was observed. Positive and negative controls were used in all experiments. The primer sequences are listed in Table 1.

### 2.4. Quantitative Analysis of the Relative Amount of *ANXA1* and *LGALS1* mRNA by Quantitative Real-Time PCR (q-PCR)

The relative quantification q-PCR assay for *ANXA1* and *LGALS1* mRNA expression was performed in an ABI Prism 7300 Sequence Detector System (Applied Biosystems, Foster City, CA, USA), according to the instructions for the SYBR Green PCR Core Reagent (Applied Biosystems), using primers specific for genes *ANXA1* and *LGALS1* [27]. Gene *ACTB* was used as endogenous control (reference gene) of the reaction, because it had shown the lowest variation compared to **α*-tubulin *and **β*2-microglobulin* genes in a previous study [28]. The primer sequences are presented in Table 1.

The expression levels of the *ANXA1*, *LGALS1*, and *ACTB *mRNA were tested in triplicate (cDNA from the same RT reaction, but in separated wells). Controls with no template cDNA were used for each assay (negative control). Samples of normal gastric mucosa were mixed to form a pool that was used as a calibrator (standard sample). The q-PCR assays were performed in a total volume of 50 *μ*L, containing 10 *μ*L of SYBR Green Master Mix (Applied Biosystems), 25 ng of cDNA, and 0.4 *μ*M of *ANXA1 *and 0.5 *μ*M* LGALS1* primers. 

After initial incubation at 50°C for 2 min to allow uracil-N-glycosylase (UNG) digestion and at 95°C for 10 min to activate the AmpliTaq Gold DNA polymerase (both provided by the Universal PCR Master Mix), the samples were amplified by subjecting them to 40 biphasic cycles of 95°C for 15 sec and 60°C for 1 min. 

The fluorescence signal was measured in the extension phase of the PCR reaction, and a threshold value (C_T_) of fluorescence in the exponential part of the amplification curve was selected. The larger the quantities of the material at start, the lower the CT values. Relative quantification (RQ) of genes *ANXA1 *and *LGALS1* was obtained as described by Pfaffl [29] and normalized with the *β-actin *control reference gene and normal gastric mucosa. The transcript levels were considered to be upregulated if RQ > 2.0.

### 2.5. Immunohistochemistry

Deparaffinized sections (4 *μ*m) were incubated in citrate buffer, pH 6.0, at 96°C for 30 minutes, washed with distilled water, incubated with 3% hydrogen peroxide in methanol (30 minutes), and washed in phosphate-buffered saline (PBS, pH 7.4). The primary antibodies rabbit polyclonal anti-Gal-1 and rabbit polyclonal anti-ANXA1 (Zymed Laboratories, Cambridge, UK) were diluted to 1 : 500 or 1 : 2000, respectively, in 1% bovine serum albumin (BSA) and applied overnight at 4°C. As negative controls, some sections were incubated with 1% BSA without any primary antibody. Fragments were then washed in PBS, incubated with the universal LSAB kit/HRP secondary antibody (Dako, USA) according to the manufacturer's protocol, washed in PBS, and developed with 3,3′-diaminobenzidine in chromogen solution (Dako, USA). The sections were washed thoroughly in distilled water, counterstained with hematoxylin and mounted on glass slides. Densitometric analysis for AnxA1 and Gal-1 immunostaining was performed using an arbitrary scale from 0 to 255 with the AxioVision software on a Zeiss-Axioskop II light microscope, and the data were expressed as mean ± SE.

### 2.6. Statistical Analysis

Fisher's exact test was used to determine if there were significant differences between groups regarding the presence of bacteria and the cagA genotype, the histological type of tumor, and the gender. The data obtained from mRNA quantification were expressed as mean ± SD. To assess differences in the mRNA relative expression levels between the groups, we used the nonparametric Mann-Whitney test. To evaluate the association between gene expression and the presence of the bacterium and cagA genotype, histological type of tumor, gender, smoking, and drinking, we used the nonparametric *t*-test with Welch's correction. The value of protein expression was expressed as mean ± SE. The mean densitometry analysis results obtained for proteins AnxA1 and Gal-1 were compared by ANOVA, followed—if significant—by the Bonferroni test. These analyses were performed using the GraphPad InStat and GraphPad Prism 4 software. The value was considered significant if *P* < 0.05.

## 3. Results

### 3.1. Molecular Diagnoses for* H. pylori-cagA*

The frequencies of cases with positive molecular diagnosis for *H. pylori* and genotype *cagA* in groups CG and GA are presented in Table 2. All 10 samples of normal mucosa were confirmed by molecular testing as *H. pylori* negative.

Out of a total of 60 samples from the case groups, 45% were *H. pylori* positive: 40% (16/40) in the CG group and 55% (11/20) in the GA group, with no significant difference between the groups (*P* = 0.29). Regarding the genotype* cagA*, 48% of *H. pylori*-positive samples were *cagA* positive: 62.5% (10/16) in the CG and 27.3% (3/11) in the GA group. Again, there was no significant difference between the groups (*P* = 0.12).

### 3.2. Relative Gene Expression Analysis

**Figure 1:**
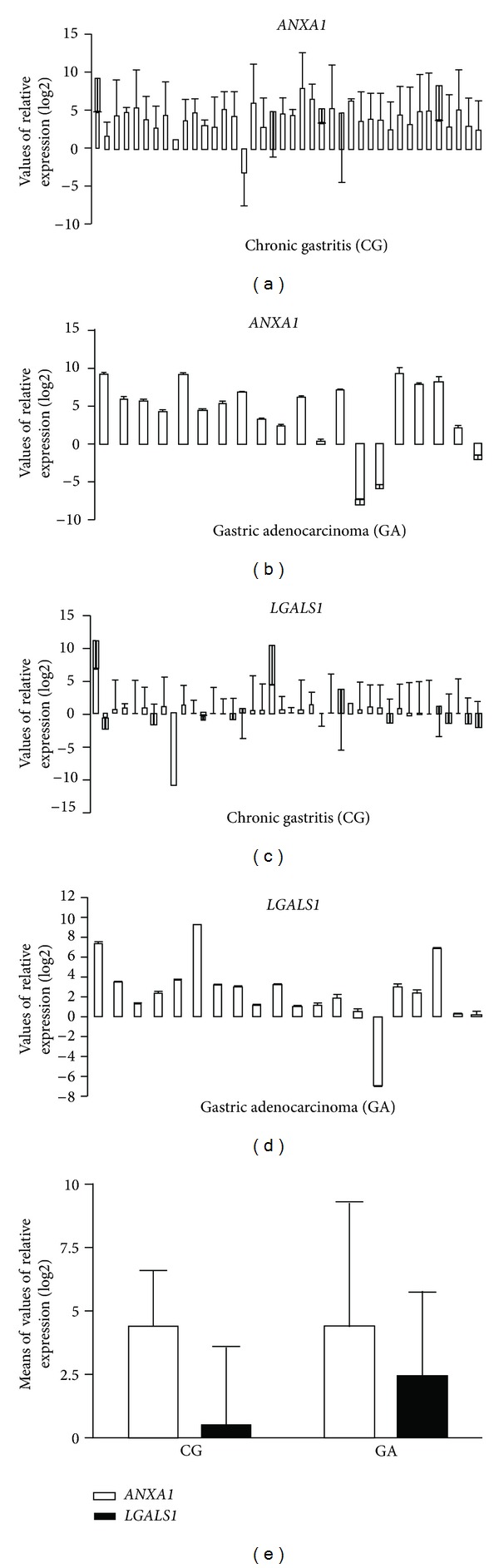
Values of relative gene expression (log2) of *ANXA1* (a, b) and *LGALS1* (c, d) and means of values of relative gene expression (e) in the chronic gastritis (CG) and gastric adenocarcinoma (GA) groups.

The relative expression levels of *ANXA1* mRNA, after normalization with the *ACTB *reference gene and comparison with the normal mucosa, were increased in 90% (36/40) of the CG cases (mean RQ = 4.26 ± 2.03) and in 80% (16/20) of the GA cases (mean RQ = 4.38 ± 4.77), so there was no statistically significant difference between the groups (*P* = 0.33). For *LGALS1*, the mRNA relative expression values found were lower; only the GA group showed overexpression in 60% (12/20) of the cases (mean RQ = 2.44 ± 3.26). The CG group showed constitutive expression (mean RQ = 0.43 ± 3.13), with only 7.5% (3/40) of the cases presenting increased expression. Thus, the mean level of *LGALS1 *mRNA expression was significantly higher in the GA than in the CG group (*P* < 0.01) (Table 3 and Figure 1).

In another analysis, we investigated the GA group for a possible association between *ANXA1* and *LGALS1* mRNA expression and risk factors such drinking, smoking, and histological type of gastric cancer (Table 4), but no association was observed. In addition, comparing the variables, gender, *H. pylori *infection, and *cagA+ *genotype, in the CG and the GA groups (Table 5), a significant difference was found in the GA group for the gender and the mean level of *ANXA1* expression (*P* = 0.04), due to a 2 times greater expression of this gene in the females than in the males of this group. Likewise, the *cagA+ *genotype also showed an association with the *ANXA1* expression level in the GA group, due to a higher mRNA expression in the *cagA-*positive compared to the *cagA-*negative cases (mean RQ 6.40 versus 2.77, *P* < 0.01). None of the other investigated factors appears to be associated with the levels of *ANXA1* and *LGALS1* mRNA.

### 3.3. Protein Expression Measured by Immunohistochemistry Assay

**Figure 2:**
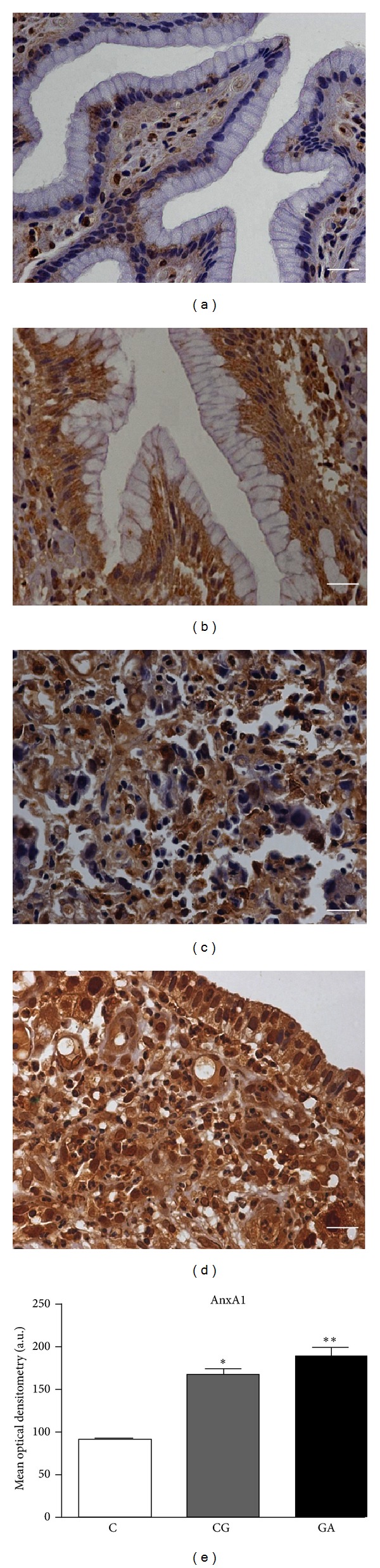
Endogenous annexin-A1 (ANXA1) expression in the gastric mucosa. Immunoreactivity of ANXA1 in sections of gastric mucosa tissue by rabbit polyclonal antibody ANXA1. (a) Histological analysis showing modest immunoreactivity in stroma and negative for epithelial cells in normal mucosa. Note the immunopositivity in basal portion of epithelial in (b) chronic gastritis and intense stromal-epithelial immunostaining in (c) diffuse-type adenocarcinoma and (d) intestinal-type adenocarcinoma. Hematoxylin counterstain. Bar: 20 *μ*m. (e) Densitometry analyses on the gastric mucosa tissues immunostained for AnxA1 in normal mucosa (C), chronic gastritis (CG), and gastric adenocarcinoma (GA) groups. Comparison between the C group and the CG (*) and GA (**) groups was made by the ANOVA test, with *P* < 0.01.

**Figure 3:**
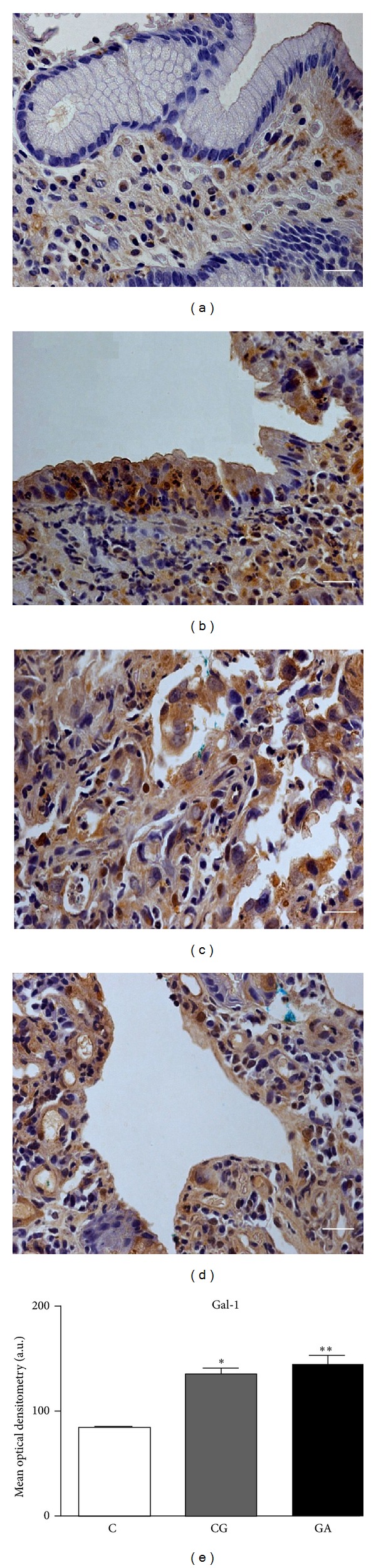
Endogenous galectin 1 (Gal-1) expression in the gastric mucosa. Immunoreactivity of Gal-1 in sections of gastric mucosa tissue by rabbit polyclonal anti-Gal-1. (a) Negative immunostaining in epithelium and modest positivity in stroma in normal mucosa. Immunopositivity in basal portion of epithelial in chronic gastritis (b) and, intense stromal-epithelial immunostaining in diffuse-type adenocarcinoma (c) and intestinal-type adenocarcinoma (d). Hematoxylin counterstain. Bar: 20 *μ*m. (e) Densitometry analyses on the gastric mucosa tissues immunostained for Gal-1 in normal mucosa (C), chronic gastritis (CG), and gastric adenocarcinoma (GA) groups. Comparison between the C group and the CG (*) and GA (**) groups was made by the ANOVA test, with *P* < 0.01.

Protein expression was evaluated in normal mucosa, CG, intestinal (IGC), and diffuse- (DGC-) type gastric cancer. Modest expression of AnxA1 and Gal-1 was observed in the stroma of normal mucosa, while the epithelium did not show any expression of these proteins (Figures 2(a) and 3(a), resp.). However, in the inflammatory process of CG mucosa, intense immunostaining of AnxA1 and Gal-1 was seen in the basal portion of the epithelium (Figures 2(b) and 3(b), resp.). Positive immunostaining for AnxA1 and Gal-1 was also observed in DGC (Figures 2(c) and 3(c), resp.) and IGC (Figures 2(d) and 3(d), resp.) tumor cells. In some areas of gastric cancer samples, it was possible to identify epithelial cells showing AnxA1 and Gal-1 expression.

The mean optical densitometry values of AnxA1 and Gal-1 expression are presented in Figures 2(e) and 3(e), respectively. The AnxA1 mean density was 90.79 for normal mucosa (C group), while for CG and GA, it was, respectively, 168.57 and 190.20. Thus, a significant difference was found comparing the normal mucosa with the CG and GA groups (*P* < 0.01 for both). The mean density of the Gal-1 protein was lower in all three groups (86.52, 136.97, and 146.30 in C, CG, and GA, resp.), showing a statistically significant difference between the normal mucosa and the CG and GA groups (*P* < 0.01 for both).

In another densitometry analysis to determine the cytoplasmic and nuclear immunoreactivity of AnxA1 separately in all groups, although observed nuclear immunostaining mainly in gastric cancer, the data obtained in our results did not show a statistically significant difference between normal mucosa and CG (*P*  =  0.19) and normal mucosa and GA (*P*  =  0.18) (data not shown).

## 4. Discussion

In the present study, we evaluated AnxA1 and Gal-1 anti-inflammatory proteins and mRNA expression in a group of precursor lesions such as chronic gastritis compared to gastric cancer and their association with risk factors. Among the risk factors, we investigated the presence of *H. pylori* in both lesions and its virulence genotype *cagA*, since this bacterium often triggers the progression of the gastric carcinogenesis cascade. To the best of our knowledge, this is the first study that has evaluated the expression of AnxA1 and Gal-1 in chronic gastritis.

We have found a high relative expression of *ANXA1* mRNA already in the CG inflammatory process, in which 90% of cases showed an increased expression level (mean  RQ  = 4.26), which became 3 to 8 times higher after normalization with the *ACTB* reference gene and comparison with normal mucosa. Similarly, in the GA group, 80% of cases presented upregulated expression levels, which were 3 to 9 times higher (mean  RQ  = 4.38) than that in normal mucosa. For *LGALS1*, our study showed a slightly increased relative expression only in 60% of the GA cases (mean  RQ  = 2.44), with an increase of 2 to 7 times, but in chronic gastritis the mean value was low (mean  RQ  = 0.43). In general, the immunohistochemical analysis confirmed the results of mRNA expression by qPCR, although in CG the relative expression levels of *LGALS1* mRNA was not equivalently increased as protein expression.

Studies on the expression of *ANXA1 *mRNA in neoplastic processes are still limited and the results are conflicting. For example, loss of expression was found in squamous cell carcinoma of the esophagus [30], prostatic adenocarcinoma [31], sinonasal adenocarcinoma [32], larynx [33], and breast cancer [34]. On the other hand, overexpression has been reported in colorectal adenocarcinoma [35], urothelial carcinoma [36], lung adenocarcinoma [37], and oral cancers [38], thus suggesting that changes in the expression levels of *ANXA1 *may be related to the tissue or tumor type. 

Martin et al. [18] reported that normal gastric mucosa shows weak expression of the protein AnxA1, while in the ulcer healing process the expression was increased, promoting the reduction of the ulcer. In contrast, Yu et al. [19] observed overexpression of both gene and protein in normal mucosa but loss of expression in 64% of primary gastric tumors, mainly correlated with advanced stage and metastasis. More recently, Zhu et al. [21] observed that AnxA1 protein is expressed in both gastric adenocarcinoma (45%) and normal tissues (69%), but with different subcellular distribution. Similar results were reported by Cheng et al. [39] that observed high AnxA expression, both mRNA and protein, associated with metastasis, invasion, and poor survival in gastric cancer patients. The authors also proposed a new mechanism of how AnxA1 regulates the gastric cancer cell invasion through activation FPR/ERK/ITGB1BP1 pathway.

In the present study, the immunohistochemical analysis for AnxA1 showed modest immunostaining in stromal cells of normal mucosa and intense expression in stroma and epithelium of both CG and GA, thus confirming the results of the mRNA expression analysis. The assay used in this study did not allow a clear differentiation of the cellular localization of the protein, although positive immunostaining was observed in the cytoplasm of epithelial cells and the nucleus of cancer cells, with lower intensity and frequency in chronic gastritis. However, when the densitometry analysis to determine cytoplasmic and nuclear immunoreactivity of AnxA1 separately was performed, the data obtained did not show a statistically significant difference among normal mucosa, chronic gastritis, and gastric adenocarcinoma. Although, there are some reports that show translocation of AnxA1 during carcinogenesis, as Alves et al. [40] that showed 87.5% positivity for AnxA1 in larynx tumors and increased immunoreactivity in the membrane compared to the cytoplasm and the nucleus. However, compared to the normal tissue, the nuclear and cytoplasmic expression was lower. In esophageal carcinoma, Liu et al. [41] found AnxA1 translocation from the cellular to the nuclear membrane. While in gastric adenocarcinoma AnxA1 showed positive nuclear staining correlated with advanced disease stage and peritoneal dissemination but in normal tissues was predominantly localized in the cytoplasm [21]. In addition, weak nuclear staining also occasionally occurred in sporadic cells of gastric tumors (14/118 cases) evaluated by Cheng et al. [39]. But, in general, the AnxA1 immunostaining was mainly cytoplasmatic in the epithelial cells.

Changes in the expression pattern of AnxA1 must be related with factors that influence its translocation and export, such as the cellular concentration of calcium, considering that, when the intracellular calcium level is increased, AnxA1 is translocated to membranes [42]. Furthermore, it is reported that protein phosphorylation is also required for this process [20]. In tumor cells, calcium and other factors may be altered, resulting in an abnormal location of AnxA1. However, this relationship needs to be better understood and may be the connection between inflammation, signal transduction, differentiation, and cellular transport in cancer [40].

To date, Gal-1 is involved in various important aspects of carcinogenesis, so mRNA and protein expressions have been examined in several types of cancer. Overexpression has been reported in colorectal cancer [43], Hodgkin's lymphoma [44], squamous cell carcinomas of the larynx, and carcinomas of the hypopharynx [45]. Conversely, there is also a report of low expression, as in chronic inflammation of nasal polyposis [27].

Two research groups evaluated the expression of Gal-1 in gastric cell lines and both agreed that this protein is overexpressed in tumor cells. Chen et al. [16] showed higher expression of the protein in TMC-1 compared to SC-M1 cells, proposing Gal-1 as a biomarker for metastasis. Lim et al. [15] investigated AGS cells infected by *H. pylori* and observed high expression levels of Gal-1 in this cell type. Recently, Estofolete et al. [46] observed by immunostaining that both Gal-1 and -3 were highly expressed in an experimental model of N-methyl-N′-nitro-N-nitrosoguanidine- (MNNG-) induced gastric carcinogenesis.

The immunohistochemistry assay performed showed modest staining for Gal-1 in the stroma of normal mucosa, while in CG and GA the staining was stronger in stroma and epithelium. In contrary, the *LGALS1* mRNA levels were lower in the CG group in comparison with the GA group. Several studies have shown lack of correlation between RNA expression and protein expression profiles using different methodologies. Some authors state that in fact the use of mRNA expression patterns is insufficient for understanding the expression of protein products, as additional posttranscriptional mechanisms, including protein translation, posttranslational modification, and degradation, may influence the level of a protein present in a given cell or tissue [47–50]. 

The comparison between the CG and GA groups regarding *ANXA1* and *LGALS1 *mRNA levels and risk factors did not show any association with the *LGALS1* mRNA levels. However, a positive association was observed between overexpression of *ANXA1* and female gender in the GA group, since in women the mRNA expression was twice as high as in men (mean RQ = 6.67 versus 3.25), and the *H. pylori-cagA+* infection showed an mRNA mean level approximately twice higher in patients infected by *cagA+* strains (mean RQ = 6.40 versus mean RQ = 2.77). Despite the relevance of the study, it should be considered some limitations due to the reduced number of cases in the subgroup stratification, which should be interpreted with cautions. It would need confirmation in further studies with larger population.

It is well known that there is sexual dimorphism in the immune and inflammatory responses in humans. Women produce more vigorous cellular and humoral reactions, are more resistant to certain infections, and suffer a higher incidence of autoimmune diseases than males [51]. It is possible that hormonal differences may explain part of this dimorphism. It has also been suggested that the *ANXA1* expression may differ in several types of cancer due to hormonal influence [52]. 

Ang et al. [52] conducted a very elegant study on the influence of AnxA1 in MCF-7 breast cancer cells, in which they showed that 17 *β*-estradiol (active metabolite of estrogen) regulates *ANXA1* expression. Moreover, 17 *β*-estradiol was shown to activate cyclic-AMP- (cAMP-) responsive element (CRE) binding (CREB) proteins to induce a transcriptional activity. The promoter region of *ANXA1* was examined and found to contain a similar, near identical, 8-nucleotide sequence (TGATGTCA) to the CRE consensus sequence (TGACGTCA). So, estrogen could also promote the transcription of *ANXA1*. Yet, those authors proposed another theory to explain the connection between estrogen and AnxA1 levels. Elevated 17 *β*-estradiol activates the ERK1/2 pathway, increasing cell proliferation, and this serves as a sign to stimulate *ANXA1* transcription in order to reduce the proliferative state. Then, AnxA1 upregulates p21, which has antiproliferative properties, and reduces the proliferation caused by the activation of ERK1/2 by estrogen. However, in our study, in the GA group the mean age of women was elevated (67.4  ±  18.4 years), which characterizes the postmenopausal phase that have decreased estrogen levels, thus not justifying the relation between increased expression of *ANXA1 *and estrogen. Therefore, further studies are needed in order to clarify this issue.

The relationship between the presence of virulence factor *CagA* and gastric carcinogenesis is well documented. Western populations infected with *CagA-*positive strains generally have an accentuated inflammatory response, with increased risk of developing peptic ulcer and stomach cancer [53]. The phosphorylation of the CagA protein activates SHP-2 (protein tyrosine phosphatase), which then inhibits the FAK (focal adhesion kinase), an enzyme that modulates adhesion, migration, and cell survival [4]. Consequently, the decline in the activity of FAK triggers a rearrangement of the cytoskeleton known as the hummingbird phenotype, characterized by elongation and spreading of host cells [54]. Furthermore, CagA participates in cell signaling by activating the *PIK3CA* and *KRAS* pathways [1], ERK (MAPK) [55, 56], and MEK/ERK and JAK1 signaling pathway in gastric cancer cells [57]. To our knowledge, there are no reports about the relationship to the CagA virulence factor and the expression of ANXA1 and Gal-1 anti-inflammatory proteins. However, Lin et al. [58] observed overexpression of AnxA4 in tumor cells of patients infected with *H. pylori* and in gastric cancer SCM-1 cells after *H. pylori* infection. Recently, Lin et al. [59] observed that infection by *H. pylori *induced a change in AnxA1 and AnxA4 localization, causing a translocation from the cytoplasm to the plasma membrane, probably for epithelial cell membrane repair in the consequence of *H. pylori*-generated membrane disruptions. So, due to the action of CagA bacterial protein in different cell signaling pathways, it is possible that it may also contribute to activation of ANXA1 expression mainly, considering that this protein plays a key role as intracellular Ca^2+^ flux, modifications of the cytoskeleton, differentiation and migration, cell growth, and apoptosis.

## 5. Conclusions

In conclusion, this study showed overexpression of both *ANXA1 *mRNA and protein already in a precursor lesion such as CG, similar to GA, in which higher expression levels were observed in *H. pylori-cagA+* cases, suggesting upregulation of this gene in early stages of gastric carcinogenesis. In turn, *LGALS1* (mRNA or protein) was slightly overexpressed in both lesions, indicating also its participation in gastric carcinogenesis. However, as in several types of cancers the role of these proteins is not yet fully understood, further investigations are needed to help clarify the molecular mechanisms by which they act in this kind of lesion.

## Figures and Tables

**Table 1:** Primers sequences used in multiplex PCR to determine *H. pylori* infection and *cagA* genotype and in q-PCR assays.

Gene	Sequence 5′–3′
*HpX *	F: CTGGAGARACTAAGYCCTCC
	R: GAGGAATACTCATTGCGAAGGCGA

*CYP1A1 *	F: CTCACCCCTGATGGTGCTAT
	R: TTTGGAAGTGCTCACAGCAG

*cagA *	F: ATGACTAACGAAACTATTGATC
	R: CAGGATTTTTGATCGCTTTATT

*ANXA1 *	F: GCAGGCCTGGTTTATTGAAA
	R: GCTGTGCATTGTTTCGCTTA

*LGALS1 *	F: GGACATCCTCCTGGACTCA
	R: GTTGAAGCGAGGGTTGAAGT

*ACTB *	F: TGCCCTGAGGCACTCTTC
	R: CGGATGTCCACGTCACAC

**Table 2:** Distribution of infection by *H. pylori* and *cagA* strains into chronic gastritis (CG) and gastric adenocarcinoma (GA) groups.

Status	CG *N* (%)	GA *N* (%)
*H. pylori *		
Positive	16 (40)	11 (55)
Negative	24 (60)	9 (45)

Total	40 (100)	20 (100)

*P*	0.29	

Genotype *cagA *		
Positive	10 (62.5)	3 (27.3)
Negative	6 (37.5)	8 (72.7)

Total	16 (100)	11 (100)

*P*	0.12	

*N*: number of individuals.

**Table 3:** Comparison of *ANXA1* and *LGALS1* mRNA relative expression levels between the chronic gastritis (CG) and the gastric adenocarcinoma (GA) groups.

Variable	*AN* *XA*1		*LG* *AL* *S*1	
	CG	GA	CG	GA
Relative expression (mean ± SD)	4.26 ± 2.03	4.38 ± 4.77	0.43 ± 3.13	2.44 ± 3.26
Minimum	−2.97	−7.88	−10.78	−6.95
Maximum	8.90	9.29	10.91	9.28
*P*	0.33		<0.01*	

*Significant difference.

**Table 4:** Relative expression of *ANXA1* and *LGALS1* mRNA in the gastric adenocarcinoma group related to drinking, smoking, and histological type of cancer.

Variable	*AN* *XA*1	*LG* *AL* *S*1
Drinking		
Yes	8 (40%)	8 (40%)
(mean ± SD)	2.29 ± 5.76	1.74 ± 1.49
No	12 (60%)	12 (60%)
(mean ± SD)	5.98 ± 2.61	2.82 ± 3.91
*P*	0.15	0.39
Smoking		
Yes	12 (60%)	12 (60%)
(mean ± SD)	4.01 ± 5.54	2.44 ± 2.18
No	8 (40%)	8 (40%)
(mean ± SD)	4.20 ± 4.17	2.45 ± 4.21
*P*	0.93	0.99
Histology		
Intestinal	12 (60%)	12 (60%)
(mean ± SD)	5.82 ± 2.89	3.60 ± 2.79
Diffuse	8 (40%)	8 (40%)
(mean ± SD)	1.55 ± 6.02	0.71 ± 3.32
*P*	0.09	0.06

**Table 5:** Relative expression of *ANXA1* and *LGALS1* mRNA in chronic gastritis (CG) and gastric adenocarcinoma (GA) groups according to gender, infection by *H. pylori*, and presence of *cagA*+ genotype.

Variable	*AN* *XA*1		*LG* *AL* *S*1	
	CG	GA	CG	GA
Gender				
Female	22 (55%)	6 (30%)	22 (55%)	6 (30%)
(mean ± SD)	4.40 ± 1.80	6.67 ± 2.00	0.29 ± 3.55	4.81 ± 3.26
Male	28 (45%)	14 (70%)	28 (45%)	14 (70%)
(mean ± SD)	4.08 ± 2.32	3.25 ± 5.16	0.60 ± 2.61	1.66 ± 2.96
*P*	0.63	0.04*	0.75	0.10
*H. pylori *				
Positive	16 (40%)	11 (55%)	16 (40%)	11 (55%)
(mean ± SD)	4.04 ± 2.59	3.91 ± 5.48	−0.05 ± 3.97	3.33 ± 3.16
Negative	24 (60%)	9 (45%)	24 (60%)	9 (45%)
(mean ± SD)	4.40 ± 1.60	4.35 ± 4.05	0.75 ± 2.46	1.36 ± 3.23
*P*	0.47	0.61	0.24	0.73
Genotype *cagA *				
Positive	10 (62.5%)	3 (27.3%)	10 (62.5%)	3 (27.3%)
(mean ± SD)	2.91 ± 2.74	6.40 ± 3.00	−1.57 ± 3.62	2.58 ± 1.26
Negative	6 (37.5%)	8 (72.7%)	6 (37.5)	8 (72.7%)
(mean ± SD)	5.49 ± 1.56	2.77 ± 6.52	1.89 ± 3.74	2.80 ± 3.10
*P*	0.14	<0.01*	0.09	0.51

*Significant difference.
